# Impact of blood pressure levels and variability after successful revascularization on the prognosis in ICAS-LVOS patients

**DOI:** 10.3389/fneur.2026.1782437

**Published:** 2026-03-05

**Authors:** Yimeng Liu, Hui Zhang, Qiang Dong, Wenjie Cao

**Affiliations:** 1 Department of Neurology, Huashan Hospital, Fudan University, Shanghai, China; 2 State Key Laboratory of Medical Neurobiology, Fudan University, Shanghai, China; 3 National Clinical Research Center for Aging and Medicine, Huashan Hospital, Fudan University, Shanghai, China

**Keywords:** blood pressure variability, endovascular treatment, intracranial atherosclerotic stenosis, large-vessel occlusive stroke, prognosis, systolic blood pressure

## Abstract

**Background:**

Optimal blood pressure management after endovascular treatment (EVT) for intracranial atherosclerosis-related large vessel occlusion stroke (ICAS-LVOS) remains uncertain. This study evaluated the impact of systolic blood pressure (SBP) and blood pressure variability (BPV) on early outcomes following successful recanalization.

**Methods:**

We prospectively enrolled 110 ICAS-LVOS patients (Jan 2020–Dec 2024). Hourly SBP was recorded for 24 h post-EVT. BPV metrics included standard deviation (SD), coefficient of variation (CV), average real variability (ARV), and fluctuation frequency (|∆SBP| ≥ 10 mmHg). Outcomes included early favorable prognosis [National Institutes of Health Stroke Scale (NIHSS) ≤ 7], early neurological deterioration (ΔNIHSS ≥ + 2), and hemorrhagic transformation (HT).

**Results:**

110 patients were recruited. Generalized estimating equation analyses demonstrated that higher hourly SBP levels and higher |∆SBP| showed an associated with a decreased likelihood of NIHSS ≤ 7 and an increased risk of ΔNIHSS ≥ + 2. Logistic regression confirmed that lower mean SBP (*p* = 0.004), maximum SBP (*p* = 0.001), SD (*p* = 0.026), CV (*p* = 0.021), and ARV (*p* = 0.018) were significantly associated with NIHSS≤7. Conversely, higher mean SBP (*p* = 0.03), max SBP (*p* = 0.016), SD (*p* = 0.017), CV (*p* = 0.014), ARV (*p* = 0.008), and |∆SBP| ≥ 10 mmHg frequency (*p* = 0.045) independently was correlated with ΔNIHSS≥ + 2. No correlation was found between BP and HT. Subgroup analyses revealed that stroke location modified the effect of ARV on NIHSS≤7 (*p* = 0.007) and mean SBP on ΔNIHSS≥ + 2 (*p* = 0.024).

**Conclusion:**

Higher post-EVT SBP levels and greater SBP variability were associated with reduced early recovery and an increased risk of neurological deterioration in ICAS-LVOS. Blood pressure should be maintained at a relatively low, consistent level.

## Introduction

Recent studies have indicated that the intensive management of blood pressure (BP) might have a detrimental effect on the prognosis of large vessel occlusive strokes (LVOS) after endovascular treatment (EVT) (1–3). However, these studies recruit predominantly strokes attributed to cardiac embolism (CE), with the exception of ENCHANTED-2/MT (the International Enhanced Control of Hypertension and Thrombolysis Stroke Study-2/Mechanical Thrombectomy), which reported 44% strokes of intracranial atherosclerosis (ICAS) in the intensive group and 53% ICAS in the control group (1). This restricted the conclusion applicability to the specific patients. Notably, patients with ICAS-LVOS exhibit distinct pathophysiological features. In this population, a higher prevalence of hypertension-related comorbidities contributing to impaired cerebral autoregulation (4), as well as long-standing stenosis inducing the augmented collateral circulation, may provide a rationale against intensive blood pressure reduction. In contrast, EVT for ICAS-LVOS often requires additional interventions such as angioplasty or stenting to optimize hemodynamic status by treating the underlying stenosis compared with LVOS attribute to embolism. In this setting, a period of rigorous blood pressure control is commonly recommended after EVT to minimize the risk of hyper perfusion and hemorrhagic transformation. Therefore, the optimal BP management strategy for ICAS-LVOS patients following EVT remains unclear. This knowledge gap highlights the need for studies specifically investigating how BP management affected the clinical outcome in this population.

Blood pressure variability (BPV) is defined as the fluctuations of BP over a period of time. A small fluctuation is essential to maintain a stable perfusion for the penumbra (5). Observational studies have demonstrated that a high level of BPV after thrombectomy is associated with symptomatic intracranial hemorrhage and poor 90-day outcomes (6–8). In the anterior circulation, patients with strokes caused by CE are more susceptible to BPV than those with intra−/extra-cranial atherosclerosis (9). Nonetheless, as for ICAS-LVOS patients, the impact of BPV after thrombectomy remained to be elucidated. Therefore, the objective of this study was to figure out how the level and the variability of BP affected the early favorable prognosis and early neurological deterioration after successful revascularization in ICAS-LVOS patients.

## Methods

### Study design and participants

A prospective cohort study of acute ischemic stroke patients admitted to hospital within 24 h since symptom onset was conducted at our institution. We screened ICAS-LVOS patients by digital subtraction angiography (DSA) from January 2020 to December 2024 in this cohort. Patients meeting the following criteria were recruited in this study: (1) adult AIS patients verified the occlusion of C6-C7 segment of internal carotid artery, V4 segment of vertebral artery, basilar artery, or M1 segment of middle cerebral arteries by DSA and undergoing EVT including thrombectomy, angioplasty and/or stenting; (2) residual fixed stenosis that measured ≥50% after several passes of thrombectomy devices; (3) TOAST classification indicating Large Artery Atherosclerosis; (4) successful revascularization defined as modified Thrombolysis in Cerebral Ischemia (mTICI) ≥ 2b (10); (5) per-hour record of blood pressure for 24 h following the operation. Patients with the evidence of CE, embolism from other sources, cryptogenic stroke, severe stenosis (≥70%) of extracranial arteries or other etiologic stenosis like vasculitis and dissection were excluded from this study.

From the recruited participants, we gathered baseline clinical data including demographics, risk factors, onset-to-door time, NIH Stroke Scale (NIHSS) scores and BP on admission. Additionally, images of Computed Tomography Perfusion (CTP) prior to EVT were post-processed using MIStar software to quantify the volume of ischemic core and penumbra. Procedural details comprising administration of intravenous thrombolysis, type of anesthesia, numbers of passes made with stent retrievers, application of angioplasty, stenting or intra-arterial tirofiban (the only GP IIb/IIIa inhibitor available in our center for intra-arterial use) and final grade of mTICI were also collected.

### Blood pressure collection

Patients were transferred to the Neurological Intensive Care Unit (NICU) for intensive monitoring for at least 24 h following EVT with the hourly measurements of BP by stroke nurses. For patients who died within 24 h, BP at 1 hour prior to death was considered as the final measurement. Missing data Patients with ≥20% missing data were excluded from the analysis. For the very few isolated missing data, last observation carried forward (LOCF) was applied. The target range of systolic blood pressure (SBP) was set <140 mmHg according to Chinese guidelines (11). Antihypertensive drugs, without restrictions, could be administrated in case of exceeding the target range. SBP level metrics consisted of mean and maximum, while systolic blood pressure variability (SPBV) metrics encompassed standard deviation (SD), coefficient of variation (CV, calculated as the ratio of SD and mean), fluctuation value ( ∣ΔSBP∣ , calculated as absolute difference between consecutive SBP measurements), frequencies of ∣ΔSBP∣ exceeding thresholds of 10 mmHg and average real variability (AVR, defined as average of ∣ΔSBP∣ ).

### Early clinical outcome

Patients were assessed with NIHSS scores 24 h after successful recanalization, which served as the early functional outcome, by certified vascular neurologists who were blinded to blood pressure data throughout the study period. Absolute NIHSS ≤7scores at 24 h after thrombectomy (24 h-NIHSS≤7) indicated early favorable prognosis. NIHSS increase ≥2scores from baseline (ΔNIHSS≥ + 2) at 24 h after thrombectomy signified early neurological deterioration. Meanwhile, non-contrast computed tomography (NCCT) was performed 24 h after EVT to determine whether intracranial hemorrhage transformation happened.

### Statistical analyses

Categorical variables were presented as frequencies (percentage), while continuous variables were presented as mean ± SD with a normal distribution, and median (interquartile range, IQR) with a non-normal distribution, evaluated by Shapiro–Wilk tests. Differences between groups for categorical variables were analyzed using Pearson’s chi-square tests or Fisher’s exact tests, while continuous variables using Student’s t tests or Mann–Whitney tests, as appropriate. Univariate and multivariate generalized estimating equations (GEE) were utilized to identify the correlation of hourly SBP and ∣ΔSBP∣ in 24 h with the outcomes. Continuous variables demonstrating non-normal distribution underwent log-transformed. Multivariable logistic regression modeling was employed for the association of SBP level and variability with the outcomes. Only factors exhibiting significance at *p* < 0.1 in univariate analyses were included in the multivariate analysis. The subgroup analysis of the association of mean SBP and AVR with prognosis was performed by multivariable logistic regression for interaction effects. All tests were two-tailed, with *p* < 0.05 considered statistically significant. All statistical analyses were performed using SPSS version 26.0.

## Results

### Baseline clinical characteristics

A total of 110 ICAS-LVOS patients with successful revascularization were recruited in the final analysis (Supplementary Figure S1). Their baseline characteristics and outcomes were summarized in Table 1. The mean age of the participants was 61.8 ± 12.4 years, with 81.8% (90/110) being male. The median NIHSS score was 13, and 68.2% (75/110) of the infarctions happened in the anterior circulation. 73.6% (81/110) and 55.5% (61/110) of patients received rescue balloon angioplasty and stent implantation, respectively. The median NIHSS score at 24 h postoperatively was 10. 44.5% (49/110) of patients achieved early favorable prognosis, 20.9% (23/110) experienced early neurological deterioration and 20.0% (22/110) suffered from intracranial hemorrhage transformation (Table 1).

**Table 1:** Baseline characteristics and clinical outcomes.

Characteristics	*n* = 110
Demographics	
Sex (male)	90 (81.8)
Age (year)	61.8 ± 12.4
BMI	25.4 (23.5–27.7)
Risk factors	
Previous stroke	24 (21.8)
Hypertension	84 (76.4)
Diabetes	37 (33.6)
Cardiovascular diseases	8 (7.3)
Smoke	57 (51.8)
Heavy use of alcohol	20 (18.2)
Details of stroke	
Onset-to-door time (hour)	5.6 (2.6–10.1)
Baseline NIHSS	13 (9–19)
Systolic blood pressure on admission (mmHg)	148.5 (134.0–168.0)
Diastolic blood pressure on admission (mmHg)	84.0 (73.0–95.0)
Location	
Anterior circulation	75 (68.2)
Posterior circulation	35 (31.8)
CTP parameters in the anterior circulation^a^	
Core(mL)	10.5 (4.0–28.0)
Penumbra(mL)	116.5 (78.0–159.4)
Ratio of low-perfusion/core	8.5 (4.5–32.2)
Reperfusion treatment	
Intravenous thrombolysis	44 (40.0)
Type of anesthesia	
General anesthesia	88 (80.0)
Local anesthesia	22 (20.0)
Numbers of passes made with stent retrievers	
0^c^	23 (20.9)
1	53 (48.2)
2	29 (26.4)
≥3	5 (4.5)
Rescue therapy	
Angioplasty	81 (73.6)
Stenting	61 (55.5)
Intraarterial tirofiban	62 (56.4)
mTICI	
2b	44 (40.0)
3	66 (60.0)
Systolic blood pressure (SBP) 24 h after endovascular treatment	
Mean SBP (mmHg)	134.3 ± 12.5
Maximum SBP (mmHg)	169.8 ± 22.1
SBP-SD (mmHg)	14.2 (11.0–18.2)
SBP-CV (%)	10.6 (8.3–13.6)
∣ΔSBP∣ >10 mmHg (n)	8 (6–11)
SBP-AVR (mmHg)	10.2 (8.0–12.9)
Early outcomes after EVT	
24 h-NIHSS	10 (3–18)
24 h-NIHSS≤7	49 (44.5)
ΔNIHSS≥ + 2	23 (20.9)
Intracranial hemorrhagic transformation	22 (20.0)
Symptomatic intracranial hemorrhage	5 (4.5)

a CT perfusion parameters were analyzed in 72 patients with anterior circulation infarction.

BMI, body mass index; NIHSS, National Institutes of Health Stroke Scale; CTP, computed tomography perfusion; mTICI, modified Thrombolysis in Cerebral Ischemia; SBP, systolic blood pressure; SD, standard deviation; CV, coefficient of variation; AVR, average real variability; EVT, endovascular treatment.

### Blood pressure and early favorable prognosis

**Figure 1:**
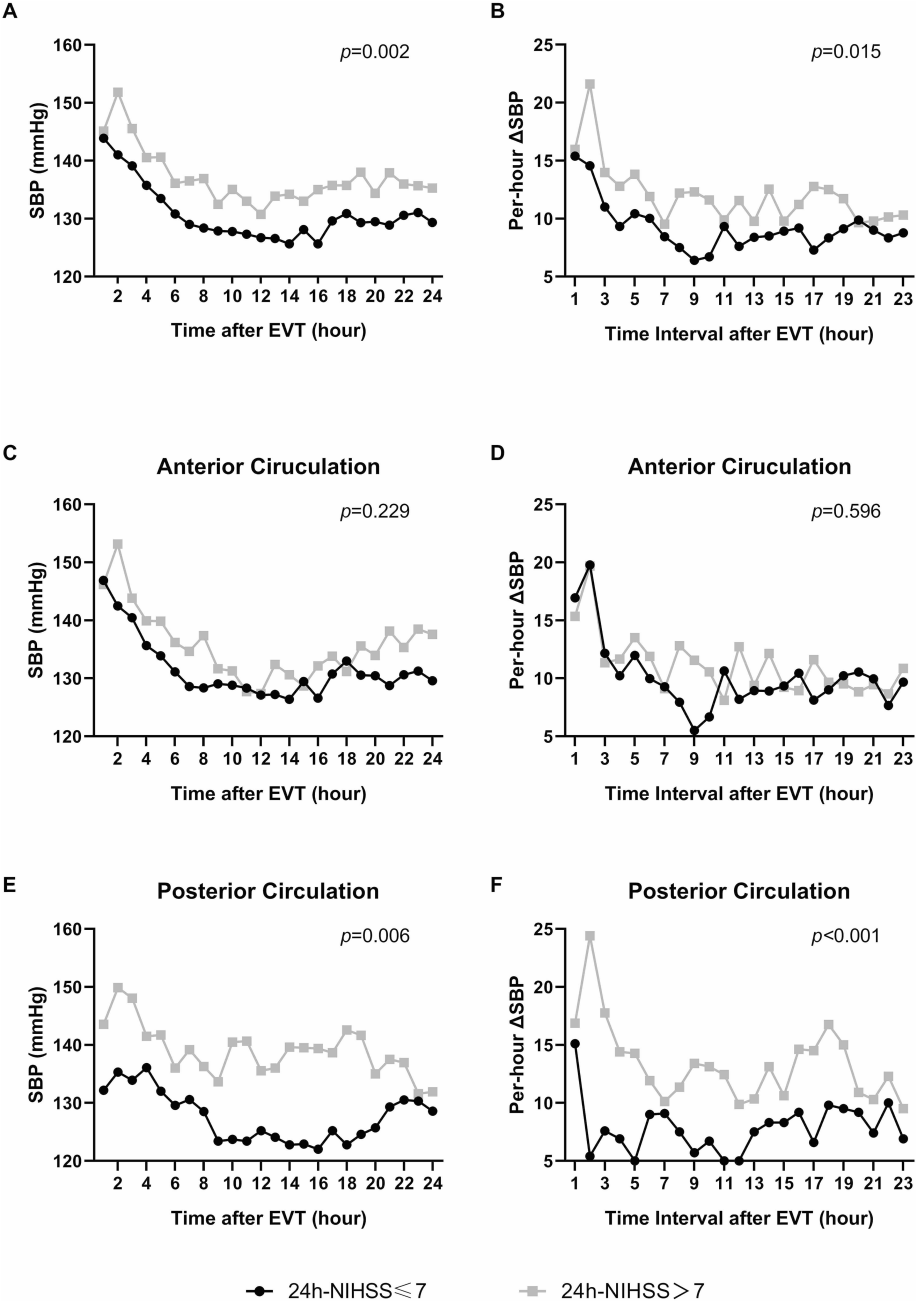
The association of per-hour SBP level and variability with early favorable prognosis 24 h after EVT (24 h-NIHSS≤7). **(A,C)** and **(E)** displayed the association of per-hour SBP level with early favorable prognosis 24 h after EVT. **(B,D)** and **(F)** displayed the association of per-hour SBP variability ( ∣ΔSBP∣ ) with early favorable prognosis. **(A)** and **(B)** The association in all the population. **(C)** and **(D)** The association in patients with anterior-circulation strokes. **(E)** and **(F)** The association in patients with posterior-circulation strokes.

In ICAS-LVOS patients, univariable GEE indicated that per-hour SBP 24 h after EVT with successful recanalization was negatively correlated with 24 h-NIHSS≤7, analyzed by correlation coefficient (*β*) as −0.017 (*p* = 0.008), indicating that higher SBP levels during this period were correlated with poorer early neurological outcomes. Baseline characteristics associated with an early favorable prognosis included a history of previous stroke (*p* = 0.045), low baseline NIHSS score (*p* < 0.001), anterior circulation infarction (*p* = 0.021), and complete revascularization (mTICI = 3) (*p* = 0.072) as well (Supplementary Table S1). After adjustment for these covariates in multivariable GEE, the association of per-hour SBP and prognosis remained statistically significant (*β*: −0.02, *p* = 0.002) (Table 2, Figures 1A,B). Compared with the patients with early favorable prognosis, those with poor prognosis had significantly higher mean SBP (137.2 *vs.* 130.7 mmHg) and maximum SBP (176.1 *vs.* 162.9 mmHg) in the first 24 h after EVT. Multivariate logistics regression also demonstrated that lower levels of mean SBP (OR: 0.943, *p* = 0.004) and peak SBP (OR: 0.965, *p* = 0.001) were independently linked with 24 h-NIHSS≤7 (Table 3).

**Table 2:** The association of per-hour SBP level and variability with early prognosis 24 h after EVT.

Variables and outcomes	Univariable analysis			Multivariable analysis		
	*β*	95%CI	*p*	*β*	95%CI	*p*
Per-hour SBP						
24hNIHSS ≤ 7^a^	−0.017	(−0.029, −0.004)	**0.008**	−0.020	(−0.033, −0.007)	**0.002**
ΔNIHSS≥ + 2^b^	0.016	(0.001, 0.030)	**0.035**	0.015	(−0.001, 0.032)	0.068
Hemorrhagic transformation	−0.010	(−0.025, 0.005)	0.179	/	/	/
Per-hour ∣ΔSBP∣						
24hNIHSS ≤ 7^c^	−0.027	(−0.040, −0.013)	**<0.001**	−0.016	(−0.029, −0.003)	**0.015**
ΔNIHSS≥ + 2^d^	0.020	(0.007, 0.034)	**0.003**	0.020	(0.008, 0.031)	**0.001**
Hemorrhagic transformation^e^	0.013	(−0.001, 0.028)	0.076	0.009	(−0.006, 0.024)	0.221

a Multivariate analysis adjusted for previous stroke, location (anterior/posterior circulation), complete revascularization (mTICI = 3) and baseline NIHSS.

b Multivariate analysis adjusted for smoke, complete revascularization and baseline NIHSS.

c Multivariate analysis adjusted for previous stroke, location (anterior/posterior circulation), complete revascularization (mTICI = 3), baseline NIHSS, mean SBP and mean pulse pressure in 24 h.

d Multivariate analysis adjusted for smoke, complete revascularization, baseline NIHSS, mean SBP and mean pulse pressure in 24 h.

e Multivariate analysis adjusted for numbers of passes made with stent retrievers, complete revascularization, baseline NIHSS, mean SBP and mean pulse pressure in 24 h.

SBP, systolic blood pressure; NIHSS, National Institutes of Health Stroke Scale; CI, confidence interval.Bold values indicate statistically significant differences (*p* <0.05).

**Table 3:** The association of SBP level and variability with early favorable prognosis and early neurological deterioration.

Variables	24 h-NIHSS ≤7 (*n* = 49)	24 h-NIHSS >7 (*n* = 61)	p	Multivariable analysis		ΔNIHSS≥ +2 (*n* = 23)	ΔNIHSS< +2 (*n* = 87)	*p*	Multivariable analysis	
				OR (95%CI)	*p*				OR (95%CI)	*p*
Mean SBP^a^	130.7 ± 12.3	137.2 ± 11.9	**0.005**	0.943 (0.907–0.981)	**0.004** ^a^	139.7 ± 14.9	132.9 ± 11.4	***0.020***	1.048 (1.005–1.093)	**0.030** ^c^
Maximum SBP	162.9 ± 21.5	176.1 ± 20.6	**0.001**	0.965 (0.944–0.986)	**0.001** ^a^	180.9 ± 23.5	166.8 ± 20.8	**0.006**	1.029 (1.005–1.053)	**0.016** ^c^
Blood pressure variability										
SBP-SD	12.0 (9.7–15.5)	17.1 (12.4–20.5)	**<0.001**	0.895 (0.811–0.987)	**0.026** ^b^	18.2 (14.2–21.8)	13.0 (10.6–17.3)	**0.003**	1.126 (1.022–1.241)	**0.017** ^d^
SBP-CV	9.2 (7.5–11.8)	11.9 (9.1–14.8)	**0.003**	0.857 (0.752–0.977)	**0.021** ^b^	12.7 (10.5–15.9)	9.8 (8.3–12.7)	**0.018**	1.180 (1.035–1.347)	**0.014** ^d^
∣ΔSBP∣ >10 mmHg	7 (5–10)	10 (7–11)	**0.002**	0.869 (0.753–1.003)	0.054^b^	10 (8–12)	8 (5–11)	**0.013**	1.191 (1.004–1.412)	**0.045** ^d^
SBP-AVR	9.0 (7.4–11.1)	11.2 (9.8–14.1)	**<0.001**	0.822 (0.699–0.967)	**0.018** ^b^	12.6 (9.7–14.7)	10.1 (7.8–11.5)	**0.005**	1.248 (1.060–1.471)	**0.008** ^d^

a Multivariate analysis adjusted for previous stroke, location (anterior/posterior circulation), complete revascularization (mTICI = 3) and baseline NIHSS.

b Multivariate analysis adjusted for previous stroke, location (anterior/posterior circulation), complete revascularization (mTICI = 3), baseline NIHSS, mean SBP and mean pulse pressure in 24 h.

c Multivariate analysis adjusted for smoke, complete revascularization and baseline NIHSS.

d Multivariate analysis adjusted for smoke, complete revascularization, baseline NIHSS, mean SBP and mean pulse pressure in 24 h.

NIHSS, National Institutes of Health Stroke Scale; OR, odds ratio; CI, confidence interval; SBP, systolic blood pressure; SD, standard deviation; CV, coefficient of variation; AVR, average real variability.Bold values indicate statistically significant differences (*p* <0.05).

In addition to the absolutely low level of SBP, variability of SBP also exerted a pivotal influence on early favorable prognosis. Higher per-hour |∆SBP| was associated with a decreased likelihood of 24 h-NIHSS ≤ 7 (univariable *β*: −0.027, *p* < 0.001; multivariable β: −0.016, *p* = 0.015). The frequencies of ∣ΔSBP∣ exceeding 10 mmHg (10 *vs.* 7, *p* = 0.002) was higher in patients with poor prognosis. What’s more, after adjusting for mean SBP, mean pulse pressure and other relevant factors, smaller SD (17.1 *vs.* 12.0 mmHg, OR, 0.895, *p* = 0.026), CV (11.9% *vs.* 9.2%, OR, 0.857, *p* = 0.021), and AVR (11.2 *vs.* 9.0 mmHg, OR, 0.822, *p* = 0.018) of SBP were independently associated with 24 h-NIHSS≤7 (Table 3).

### Blood pressure and early neurological deterioration

**Figure 2:**
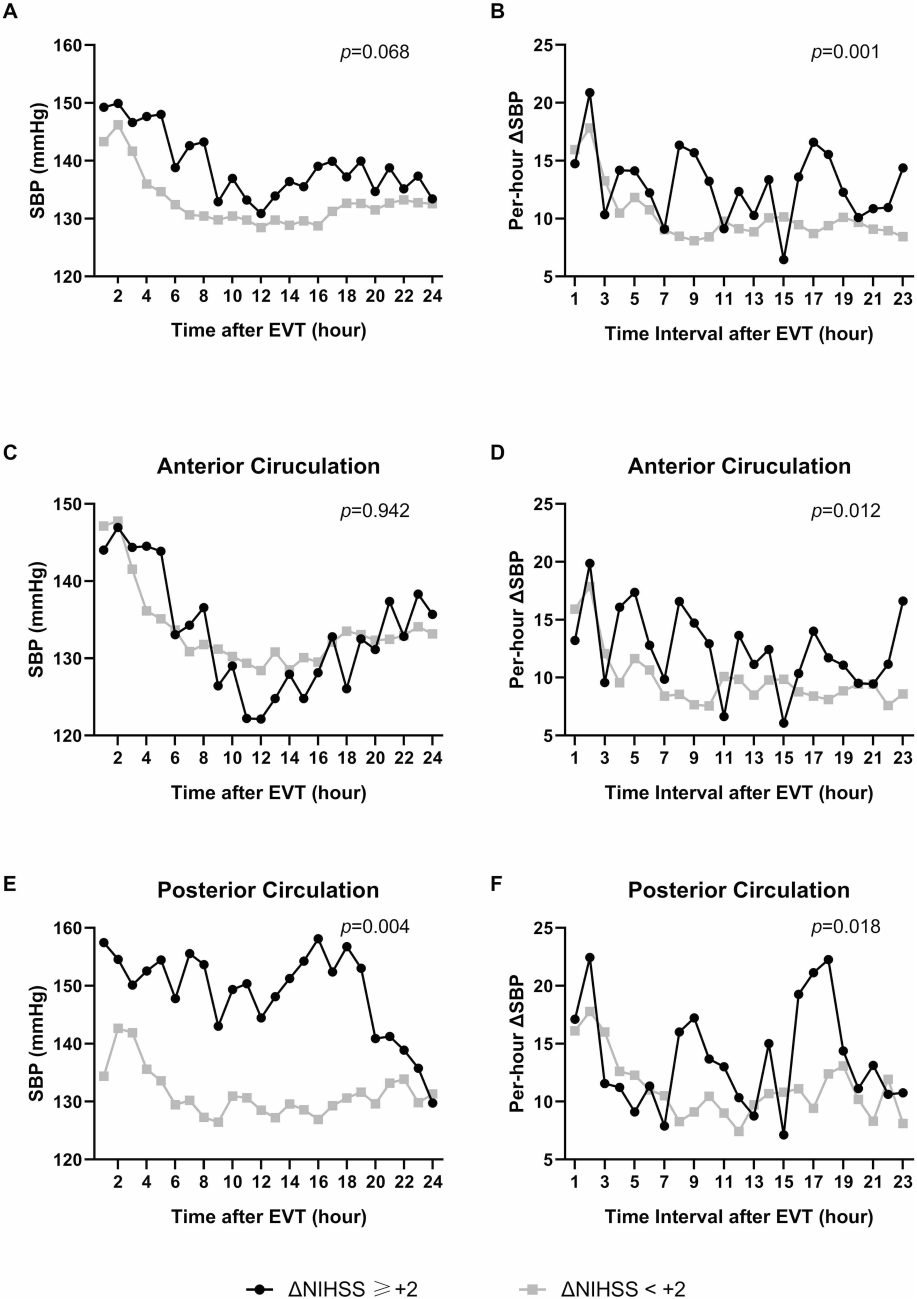
The association of per-hour SBP level and variability with early neurological deterioration 24 h after EVT (ΔNIHSS≥ + 2). **(A,C)** and **(E)** displayed the association of per-hour SBP level with early neurological deterioration 24 h after EVT. **(B,D)** and **(F)** displayed the association of per-hour SBP variability ( ∣ΔSBP∣ ) with early neurological deterioration. **(A)** and **(B)** The association in all the population. **(C)** and **(D)** The association in patients with anterior-circulation strokes. **(E)** and **(F)** The association in patients with posterior-circulation strokes.

For the outcome of early neurological deterioration, significantly distinct baseline factors contain incomplete revascularization (mTICI = 2b) (*p* = 0.006), lower baseline NIHSS score (*p* = 0.048) and smoke (*p* = 0.066) (Supplementary Table S2). Univariable GEE revealed hourly SBP (*β*: 0.016, *p* = 0.035) after EVT is positively correlated with ΔNIHSS≥ + 2, indicating that higher SBP levels during this period were correlated with early neurological deterioration. However, this correlation was diminished in multivariate analysis (*β*: 0.15, *p* = 0.068) (Table 2, Figures 2A,B). Nevertheless, univariable and multivariable logistics regression consistently indicated that higher mean SBP (139.7 *vs.* 132.9 mmHg, OR: 1.048, *p* = 0.030) and maximum SBP (180.9 vs. 166.8 mmHg, OR: 1.029, *p* = 0.016) were independently associated with ΔNIHSS≥ + 2 (Table 3).

With regard to BPV, multivariable analysis elucidated that hourly ∣ΔSBP∣ (*β*: 0.020, *p* = 0.001), frequencies of ∣ΔSBP∣ exceeding 10 mmHg (10 *vs.* 8, OR: 1.191, *p* = 0.045), SD (18.2 *vs.* 13.0 mmHg, OR: 1.126, *p* = 0.017), CV (12.7% *vs.* 9.8%, OR: 1.180, *p* = 0.014), and AVR (12.6 *vs.* 10.1 mmHg, OR: 1.248, *p* = 0.008) of SBP exhibited an independent correlation with an increased risk of. ΔNIHSS≥ + 2 (Table 3).

### Blood pressure and intracranial hemorrhage transformation

It turned out that there is no significant correlation between SBP levels (univariable *p* = 0.179) or variability (univariable *β*: 0.013, *p* = 0.076; multivariable *p* = 0.585) in 24 h after EVT with intracranial hemorrhage transformation (Table 2).

### Subgroup analyses

To further evaluate the robustness and internal consistency of the impact of SBP level and variability on the early prognosis, subgroup analyses were performed with consideration of history of hypertension, location of stroke, mTICI grade of recanalization, angioplasty, stenting, SBP on admission, core of infarction and baseline NIHSS at admission. Subsequently, we observed that location of stroke (p for interaction: 0.007) and SBP on admission (p for interaction: 0.034) modified the effect of AVR on 24 h-NIHSS≤7 (Supplementary Table S4). Meanwhile, location of stroke (p for interaction: 0.024) and baseline NIHSS (p for interaction: 0.026) demonstrated to modify the effect of mean SBP on ΔNIHSS≥ + 2 (Supplementary Table S5). For patients with ICAS-LVOS in the posterior circulation, higher SBP variability (AVR) was linked to a decreased likelihood of 24 h-NIHSS ≤ 7 (OR: 0.363, *p* = 0.047), and higher mean SBP was linked to an increased risk of ΔNIHSS ≥ + 2 (OR: 1.112, *p* = 0.040) (Supplementary Tables S4, S5). Figures 1C–F and Figures 2C–F showed the associations of per-hour SBP and ∣ΔSBP∣ in the anterior and posterior circulation, respectively. It suggested the potential benefits of lower levels of SBP and BPV in the posterior circulation subgroup, which requires further validation.

## Discussion

This observational study substantiated that higher hourly SBP levels and higher mean SBP within 24 h after EVT with successful recanalization were associated with a decreased likelihood of 24 h-NIHSS ≤ 7 and an increased risk of ΔNIHSS ≥ + 2. Greater SBP variability (including SD, CV, |∆SBP|, frequency of |∆SBP| > 10 mmHg, and AVR) was uniformly associated with a decreased likelihood of 24 h-NIHSS ≤ 7 and an increased risk of ΔNIHSS ≥ + 2. The significant association of SBP and intracranial hemorrhage transformation was not observed in ICAS-LVOS patients in our study. Notably, SBPV remained statistically significant after adjusting for mean SBP and pulse pressure, indicating its independent correlation with clinical outcomes. Additionally, this effect appeared to be modified by posterior circulation location, although it was based on a small subgroup with limited sample size. Our study clarified the impact of postoperative BP on early clinical outcomes in patients with ICAS-LVOS, prioritizing investigation into BP management in ICAS-LVOS patients.

ENCHANTED-2/MT, as mentioned above, was conducted in China with nearly half of participants attributed to ICAS, who had large penumbra (83 mL in the intensive group while 84 mL in the regular group) (1). The SBP target for the intensive management group in this study was 120 mmHg, which was proved to do harm to clinical outcomes, suggesting that SBP should avoid less than 120 mmHg. The mean SBP in our study was 134.3 ± 12.5 mmHg, aligning with the regular management group in ENCHANTED-2/MT. Our study, based on the real-world clinical data, suggested that relatively low level of SBP is related to early good prognosis while high level is related to deterioration on the condition of non-intensive BP control. Previous opinion believed that the relationship of BP after stroke and prognosis conformed to a *U*-shaped curve (4), which means either excessively high or low level of BP would have an adverse influence. SBP threshold associated with good prognosis was 158 mmHg confirmed by the BEST study (12).

For LVOS patients, at the onset of occlusion, BP gradients promote and maintain collateral circulation open to compensate the perfusion for penumbra. BP increases automatically as a result. At this stage, higher blood pressure levels are associated with better performance of collateral circulation and smaller baseline infarct cores (13). With successful recanalization by EVT, cerebral perfusion recovered, accompanied by decrease of blood pressure. Compared with patients who failed to recanalization, those with successful recanalization experienced earlier and faster stabilization of blood pressure (14). Higher level of SBP was correlated with less 90-day functional independence for these patients (15). In addition to the extent of reperfusion, collateral also influenced prognosis. A registry study illustrated that the significant association of high SBP and prognosis only observed in anterior LVOS patients with poor collateral circulation (16). In general, ICAS-LVOS patients have better collateral circulation than other causes of LVOS, and might less influenced by blood pressure level. A post-hoc analysis of the OPTIMAL-BP study confirmed no significant differences in prognosis between intensive (mean: 128.4 mmHg) and conventional (mean: 140.0 mmHg) BP management (17). However, this study only included 59 ICAS-LVOS in the anterior circulation. Our study recruited a larger number of cases and included patients with posterior circulation ICAS-LVOS. After adjusting for the location of stroke, it turned out that relatively low level of SBP was associated with 24 h-NIHSS≤7, a sign of early favorable prognosis, and high level with ΔNIHSS≥ + 2, a sign of early deterioration. Additionally, the association is more significant in the posterior circulation stroke, consequently, it remained necessary for ICAS-LVOS patients to maintain SBP in a relatively lower range in consideration of location and collateral.

The association of BPV and poor outcome after EVT has been clarified (6–8, 18). However, these studies focused primarily on strokes caused by CE in the western population. It remains unclear how BPV influence the prognosis of ICAS-LVOS. A retrospective study figured out that high level of BPV is significantly correlated with 90-day functional independence in CE-LVOS patients, instead of LAA-LVOS (9). This study only recruited stroke patients in the anterior circulation and failed to adjust the influence of absolute value of BP. Besides, extracranial stenosis occupied above half of LAA patients. Another observational study found that high level of BPV was associated with infarct core growth and poor prognosis in LVOS patients with good baseline perfusion. Good baseline perfusion was defined by a hypoperfusion intensity ratio (HIR) > 0.5 (volume of Tmax > 10 s / volume of Tmax > 6 s), which also showed high specificity for ICAS-LVOS. On the contrary, the association disappear in patients with HIR < 0.5 (19). Our study confirmed that small range of SBPV related to early favorable prognosis, especially in the posterior circulation, while large range of SBPV related to early deterioration.

All the current trials concentrated on reach the ideal SBP range and ignored BPV since monitoring and controlling BPV remains a substantial challenge. Non-invasive methods include traditional per-hour record of BP (hour-to-hour BPV) and newly-emerged near-infrared sensors to calculate beat to beat-to-beat BPV. It has been proved that beat-to-beat BPV measured for just 5 min after EVT could predict the 90-day poor outcome (20, 21). Besides, achieving personal cerebral blood flow autoregulation ranges by near-infrared spectroscopy or transcranial Doppler can help establish individualized BP target (16, 22), to maintain stable blood flow and attenuate BPV. Additionally, in the view of antihypertensive drugs, calcium channel blockers and diuretics were capable of reducing BPV, compared with *β*-receptor blockers (23). However, these findings were implied by *post hoc* analyses, requiring further clinical validation.

Our study selected the NIHSS score at 24 h after recanalization as the primary outcome instead of 90-day modified Rankin Scale (mRS), adopted by most prognostic studies in stroke. Recently, there is growing evidence examining the potential of 24-h NIHSS after EVT as an alternative endpoint (24–26). The outcome is obtained by face-to-face physical examination rather than telephone evaluation of mRS. Furthermore, this endpoint can avoid loss to follow-up and is less susceptible to interference of complications or comorbidities that may disturb functional assess. Previous studies have borne out that NIHSS≤7 at 24 h could predict 90-day mRS 0–2 (24).

There are several limitations in our study. Firstly, this is an observational study from real world. A target for BP control (SBP < 140 mmHg,) was determined under the guidance, resulting a mild difference in the absolute value of SBP in two groups. Further exploration should be taken for BP management target. Secondly, most hemorrhages were diagnosed after the 24-h BP monitoring window, except for 5 symptomatic cases in which BP was reduced more aggressively following hemorrhage. Likewise, intraprocedural contrast staining often prompted more intensive blood pressure reduction, introducing potential bias. These real-world confounders should be considered when interpreting our findings. Additionally, our study only analyzed the functional outcome, neglecting radiological outcome such as images of follow-up perfusion or final infarction. Furthermore, The proportion of patients undergoing rescue stenting in our cohort (55.5%) is relatively high, though comparable to other studies (e.g., 47.7% (27); 37.8% (28)). The interaction between stenting and blood pressure on outcomes was not significant (p for interaction > 0.05, Supplementary Tables S4, S5), suggesting no substantial confounding. However, we cannot exclude the possibility that the high stenting rate influenced blood pressure requirements. Further studies are needed to clarify this interplay from the point of hemodynamics. Last but not least, the viewpoint that prognosis in the posterior circulation was more sensitive to SBP level and variability was demonstrated by subgroup analysis with limited sample size, which should be validated in large-scale observational studies or clinical trials.

## Conclusion

In summary, this observational study led to the conclusion that higher 24-h SBP levels and variability were negatively associated with early favorable prognosis and positively associated with early neurological deterioration in ICAS-LVOS patients with successful revascularization. No significant association was found between BP and intracranial hemorrhage transformation. Although intensive BP management precipitate poor prognosis, it is advisable to control the BP in a relatively low range, beyond this, it is critical to pay attention to drugs and therapies that can diminish BPV, contributing to maintain cerebral perfusion stable and optimize functional prognosis.

## Data Availability

The raw data supporting the conclusions of this article will be made available by the authors, without undue reservation.
