# Supplementary material for: Impact of blood pressure levels and variability after successful revascularization on the prognosis in ICAS-LVOS patients
Source: Front Neurol. 2026 Mar 5;17:1782437. doi: 10.3389/fneur.2026.1782437 (PMC12999924; doi:10.3389/fneur.2026.1782437)
Supplement: Supplementary file 1 [file Data_Sheet_1.pdf]

Impact of Blood Pressure Levels and Variability After Successful Revascularization on the prognosis in ICAS-LVOS Patients

Supplemental Material

Figure S1 Study Flowchart.

Table S1 Baseline characteristics and treatment details of 24h-NIHSS≤7.

Table S2 Baseline characteristics and treatment details of ΔNIHSS<+2.

Table S3 Baseline characteristics and treatment details of intracranial hemorrhage transformation.

Table S4 Subgroup analysis of the association of mean SBP and AVR with early favorable prognosis (24h-NIHSS≤7).

Table S5 Subgroup analysis of the association of mean SBP and AVR with early neurological deterioration (ΔNIHSS≥+2).

Figure S1 Study Flowchart.

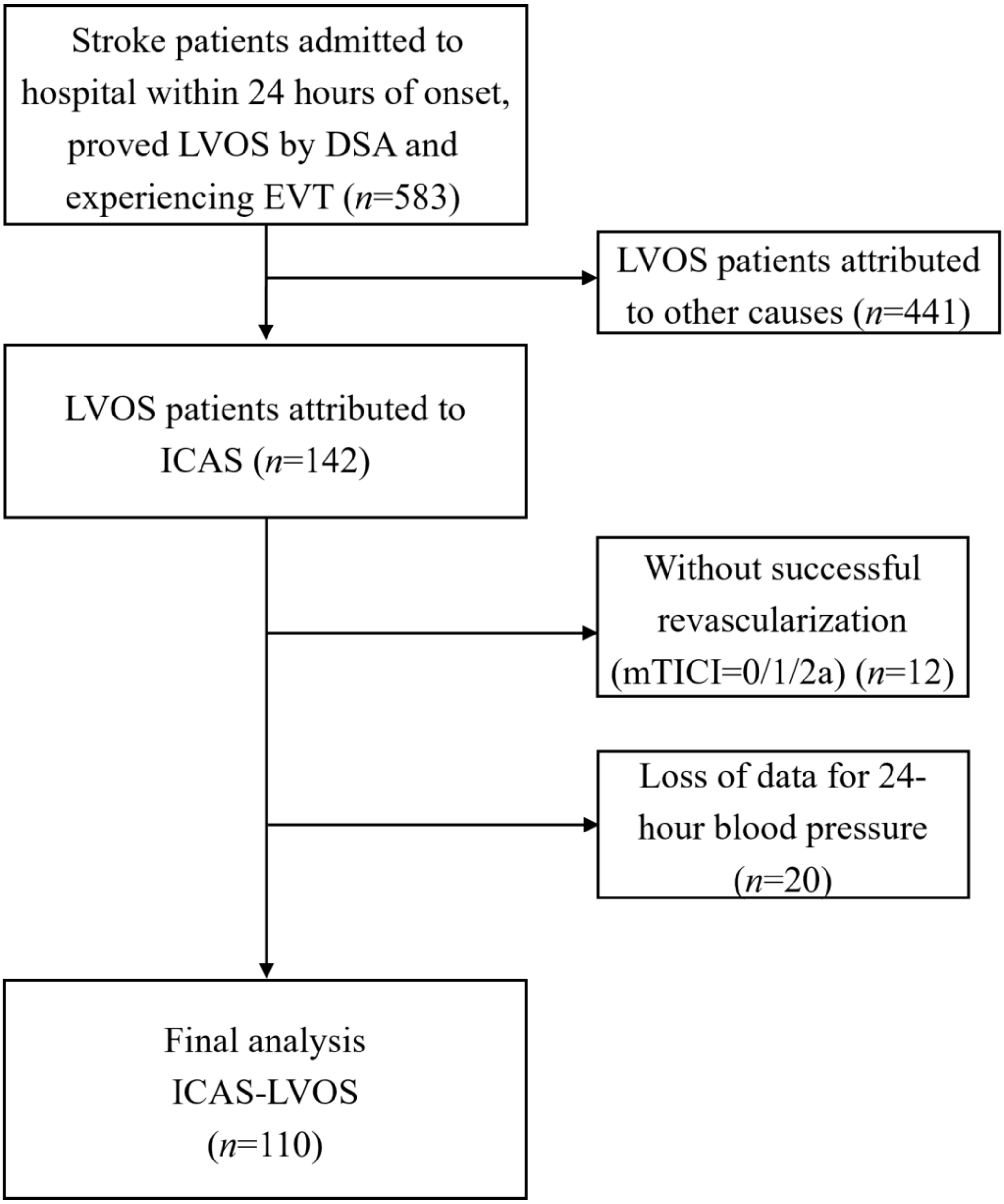

Abbreviations:

LVOS = large-vessel occlusive stroke, DSA = digital subtraction angiography, EVT = endovascular treatment, ICAS = intracranial atherosclerotic stenosis; mTICI = modified Treatment In Cerebral Infarction.

Table S1 Baseline characteristics and treatment details of 24h-NIHSS≤7.

	**24h-NIHSS≤7 (*n*=49)**	**24h-NIHSS>7 (*n*=61)**	**p**
**Demographics**			
Sex (male)	41 (83.7）	49 (80.3)	0.651
Age (year)	60.8±11.1	62.6±13.4	0.458
BMI	25.25 (22.86–27.33)	25.56 (23.88–27.77)	0.482
**Risk factors**			
Previous stroke	15 (30.6)	9 (14.8)	**0.045**
Hypertension	36 (73.5)	48 (78.7）	0.522
Diabetes	16 (32.7)	21 (34.4)	0.845
Cardiovascular diseases	4 (8.2)	4 (6.6)	1.000
Smoke	29 (59.2)	28 (45.9)	0.166
Heavy use of alcohol	10 (20.4)	10 (16.4)	0.587
**Details of stroke**			
Onset-to-door time (hour)	6.0 (2.8–10.4)	5.0 (2.6–9.5)	0.866
Baseline NIHSS	10 (7–15)	17 (12–23)	**<0.001**
SBP on admission (mmHg)	148.0 (136.0–162.0)	149.0 (131.0–168.0)	0.757
DBP on admission (mmHg)	86.0 (77.0–96.0)	80.0 (70.0–92.0)	0.143
Anterior stroke	39 (79.6)	36 (59.0)	**0.021**
CTP perfusion in the anterior circulation [a]			
Core (mL)	8.0 (3.0–25.0）	17.5 (5.0–33.0)	0.106
Penumbra (mL)	119.0 (80.0–160.0)	106.5 (78.0–135.0)	0.596
Ratio of low-perfusion/core	12.3 (5.1–33.6)	5.6 (4.0–27.8)	0.138
**Reperfusion treatment**			
Intravenous thrombolysis	23 (46.9)	21 (34.4)	0.183
General anesthesia	12 (24.5)	10 (16.4)	0.291
Numbers of passes made with stent retrievers			0.185
0	12 (24.5)	11 (18.0）	
1	25 (51.0)	28 (45.9)	
2	12 (24.5)	17 (27.9)	
≥3	0 (0)	5 (8.2)	
Rescue therapy			
Angioplasty	36 (73.5)	45 (73.8)	0.972

	**24h-NIHSS≤7 (*n*=49)**	**24h-NIHSS>7 (*n*=61)**	**p**
Stenting	24 (49.0)	37 (60.7)	0.221
Intraarterial tirofiban	27 (55.1)	35 (57.4)	0.811
Complete recanalization (mTICI =3)	34 (69.4)	32 (52.5)	0.072

[a] CT perfusion parameters were analyzed in 72 patients with anterior circulation infarction.

Abbreviations: BMI = body mass index, NIHSS = National Institutes of Health Stroke Scale, SBP = systolic blood pressure, DBP= diastolic blood pressure, CTP = computed tomography perfusion, mTICI = modified Treatment In Cerebral Infarctin.

Table S2 Baseline characteristics and treatment details of ΔNIHSS<+2.

	**ΔNIHSS≥+2 (*n*=23)**	**ΔNIHSS<+2 (*n*=87)**	**p**
**Demographics**			
Sex (male)	19 (82.6)	71 (81.6)	1.000
Age (year)	60.5±13.2	62.1±12.3	0.587
BMI	25.10 (22.92–27.36)	25.43 (23.58–27.78)	0.625
**Risk factors**			
Previous stroke	3 (13.0)	21 (24.1)	0.252
Hypertension	18 (78.3)	66 (75.9)	0.810
Diabetes	10 (43.5)	27 (31.0)	0.261
Cardiovascular diseases	2 (8.7)	6 (6.9)	1.000
Smoke	8 (34.8)	49 (56.3)	0.066
Heavy use of alcohol	2 (8.7)	18 (20.7)	0.307
**Details of stroke**			
Onset-to-door time (hour)	4.2 (2.8–9.6)	5.7 (2.7–10.1)	0.971
Baseline NIHSS	11 (7–16)	14 (10–19)	**0.048**
SBP on admission (mmHg)	155.0 (138.5–180.0)	148.0 (133.5-152.5)	0.460
DBP on admission (mmHg)	84.0 (69.0–94.0)	85.0 (74.0–95.0)	0.581
Anterior stroke	14 (60.9)	61 (70.1)	0.397
CTP perfusion in the anterior circulation [a]			
Core (mL)	15.5 (5.0–51.0)	10.0 (3.0–27.0)	0.267
Penumbra (mL)	113.0 (63.0–189.0)	116.5 (80.0–158.0)	0.977
Ratio of low-perfusion/core	4.7 (3.3–27.4)	11.4 (4.8–34.0)	0.130
**Reperfusion treatment**			
Intravenous thrombolysis	9 (39.1)	35 (40.2)	0.924
General anesthesia	20 (87.0)	68 (78.2)	0.519
Numbers of passes made with stent retrievers			0.317
0	6 (26.1)	17 (19.5)	
1	12 (52.2)	41 (47.1)	
2	3 (13.0)	26 (29.9)	
≥3	2 (8.7)	3 (3.4)	
Rescue therapy			
Angioplasty	12 (65.2)	66 (75.9)	0.303

	ΔNIHSS≥+2 (*n*=23)	ΔNIHSS<+2 (*n*=87)	p
Stenting	13 (56.5)	48 (55.2)	0.908
Intraarterial tirofiban	11 (47.8)	51 (58.6)	0.353
Complete recanalization (mTICI =3)	8 (34.8)	58 (66.7)	**0.006**

[a] CT perfusion parameters were analyzed in 72 patients with anterior circulation infarction.

Abbreviations: BMI = body mass index, NIHSS = National Institutes of Health Stroke Scale, SBP = systolic blood pressure, DBP= diastolic blood pressure, CTP = computed tomography perfusion, mTICI = modified Treatment In Cerebral Infarction.

Table S3 Baseline characteristics and treatment details of intracranial hemorrhage transformation.

	Hemorrhage transformation (*n*=22)	NO hemorrhage transformation (*n*=88)	p 值
**Demographics**			
Sex (male)	20 (90.9)	70 (79.5)	0.354
Age (year)	60.8±15.1	62.0±11.7	0.686
BMI	25.26 (23.18–25.95)	25.43 (23.53–27.78)	0.312
**Risk factors**			
Previous stroke	5 (22.7)	19 (21.6)	1.000
Hypertension	14 (63.6)	70 (79.5)	0.116
Diabetes	7 (31.8)	30 (34.1)	0.840
Cardiovascular diseases	2 (9.1)	6 (6.8)	1.000
Smoke	11 (50.0)	46 (52.3)	0.849
Heavy use of alcohol	4 (18.2)	16 (18.2)	1.000
**Details of stroke**			
Onset-to-door time (hour)	5.5 (2.5–9.8)	5.5 (2.7–10.2)	0.982
Baseline NIHSS	17.5 (10–37)	12 (9–18)	0.078
SBP on admission (mmHg)	153.0 (134.0–178.0)	148.0 (134.0–162.5)	0.348
DBP on admission (mmHg)	84.5 (75.0–89.0)	83.5 (72.0–96.5)	0.435
Anterior stroke	13 (59.1)	62 (70.5)	0.306
CTP perfusion in the anterior circulation [a]			
Core (mL)	20.0 (6.0–61.0)	9.0 (4.0–25.0)	0.124
Penumbra (mL)	131.0 (78.0–182.0)	113.0 (80.0–153.0)	0.519
Ratio of low-perfusion/core	4.8 (3.3–14.0)	11.7 (4.8–32.2)	0.139
**Reperfusion treatment**			
Intravenous thrombolysis	6 (27.3)	38 (43.2)	0.173
General anesthesia	18 (81.8)	70 (79.5)	1.000
Numbers of passes made with stent retrievers			**0.046**
0	0 (0)	23 (26.1)	
1	13 (59.1)	40 (45.5)	
2	7 (31.8)	22 (25.0)	
≥3	2 (9.1)	3 (3.4)	

	**Hemorrhage transformation (*n*=22)**	**NO hemorrhage transformation (*n*=88)**	**p 值**
Rescue therapy			
Angioplasty	16 (72.7)	65 (73.9)	0.914
Stenting	10 (45.5)	51 (58.0)	0.291
Intraarterial tirofiban	10 (45.5)	52 (59.1)	0.249
Complete recanalization (mTICI =3)	8 (36.4)	58 (65.9)	**0.011**

[a] CT perfusion parameters were analyzed in 72 patients with anterior circulation infarction.

Abbreviations: BMI = body mass index, NIHSS = National Institutes of Health Stroke Scale, SBP = systolic blood pressure, DBP= diastolic blood pressure, CTP = computed tomography perfusion, mTICI = modified Treatment In Cerebral Infarctin.

Table S4 Subgroup analysis of the association of mean SBP and AVR with early favorable prognosis (24h-NIHSS≤7).

	**Mean SBP**				**24hAVR**			
	**OR**	**95%CI**	**p**	**p for interaction**	**OR**	**95%CI**	**p**	**p for interaction**
Hypertension				0.089				0.780
Yes	0.924	0.876-0.974	0.003		0.852	0.718-1.011	0.067	
No	1.050	0.926-1.192	0.445		0.254	0.034-1.912	0.183	
Location				0.081				**0.007**
Anterior circulation	0.969	0.921-1.018	0.211		0.996	0.812-1.222	0.973	
Posterior circulation	0.913	0.842-0.991	0.029		0.363	0.134-0.988	0.047	
Recanalization				0.661				0.470
mTICI=2b	0.937	0.873-1.005	0.070		0.776	0.556-1.083	0.136	
mTICI=3	0.942	0.894-0.991	0.022		0.883	0.723-1.077	0.219	
Angioplasty				0.448				0.997
Yes	0.939	0.897-0.983	0.007		0.823	0.670-1.011	0.064	
No	0.953	0.853-1.066	0.402		0.766	0.515-1.140	0.189	
Stenting				0.999				0.138
Yes	0.940	0.888-0.994	0.031		0.687	0.511-0.924	0.013	
No	0.932	0.873-0.995	0.036		0.915	0.705-1.188	0.507	
SBP on admission				0.766				**0.034**
≥160mmHg	0.936	0.859-1.020	0.134		1.013	0.779-1.317	0.922	
<160mmHg	0.937	0.890-0.986	0.013		0.646	0.482-0.866	0.003	
Core				0.196				0.332
≥15ml	0.972	0.923-1.024	0.292		0.865	0.659-1.137	0.299	
<15ml	0.919	0.860-0.982	0.012		0.765	0.594-0.985	0.038	
Baseline NIHSS				0.865				0.912
≤15	0.940	0.894-0.988	0.016		0.851	0.682-1.061	0.152	
>15	0.932	0.855-1.016	0.110		0.876	0.666-1.153	0.345	

Table S5 Subgroup analysis of the association of mean SBP and AVR with early neurological deterioration (ΔNIHSS≥+2).

	Mean SBP				24hAVR			
	**OR**	**95%CI**	**p**	**p for interaction**	**OR**	**95%CI**	**p**	**p for interaction**
Hypertension				0.079				0.350
Yes	1.072	1.017-1.131	0.010		1.371	1.098-1.713	0.005	
No	0.990	0.888-1.105	0.863		0.987	0.727-1.340	0.934	
Location				**0.024**				0.925
Anterior circulation	0.998	0.945-1.053	0.932		1.260	1.006-1.579	0.044	
Posterior circulation	1.112	1.005-1.232	0.040		1.365	0.874-2.131	0.171	
Recanalization				0.209				0.599
mTICI=2b	1.025	0.972-1.080	0.362		1.218	1.007-1.473	0.042	
mTICI=3	1.084	1.009-1.165	0.028		1.376	0.954-1.985	0.087	
Angioplasty				0.933				0.755
Yes	1.047	0.997-1.101	0.067		1.273	1.038-1.560	0.020	
No	1.104	0.975-1.250	0.120		1.253	0.904-1.739	0.176	
Stenting				0.521				0.926
Yes	1.038	0.976-1.104	0.232		1.207	0.961-1.517	0.106	
No	1.055	0.990-1.124	0.100		1.231	0.932-1.626	0.143	
SBP on admission				0.516				0.561
≥160mmHg	1.071	0.992-1.157	0.080		1.224	0.907-1.652	0.186	
<160mmHg	1.035	0.976-1.098	0.247		1.305	1.044-1.631	0.019	
Core				0.079				0.391
≥15ml	1.005	0.950-1.064	0.865		1.374	1.007-1.874	0.045	
<15ml	1.090	1.019-1.165	0.012		1.409	1.050-1.890	0.022	
Baseline NIHSS				0.026				0.125
≤15	1.087	1.025-1.153	0.006		1.460	1.076-1.982	0.015	
>15	0.973	0..900-1.053	0.496		1.187	0.959-1.468	0.115	
